# Risk Values of Weight and Body Mass Index for Chest Wall Thickness in Patients Requiring Needle Thoracostomy Decompression

**DOI:** 10.1155/2020/2070157

**Published:** 2020-10-26

**Authors:** Chia-Hung Hsu, Tzu-Yin Lin, Ju-Chi Ou, Jiann Ruey Ong, Hon-Ping Ma

**Affiliations:** ^1^Department of Emergency Medicine, Shuang-Ho Hospital, Taipei Medical University, Taipei, Taiwan; ^2^Graduate Institute of Prevention and Injury Control, Taipei Medical University, Taipei, Taiwan; ^3^Department of Family Medicine, Shuang-Ho Hospital, Taipei Medical University, Taipei, Taiwan; ^4^Department of Emergency Medicine, School of Medicine, Taipei Medical University, Taipei, Taiwan

## Abstract

**Introduction:**

Emergency decompression is needed in patients with tension pneumothorax, a life-threatening condition. The catheter-based needle thoracostomy was suggested using a 5 cm catheter inserted into the 2nd intercostal space (ICS) and 5th ICS according to the ninth and tenth editions of Advanced Trauma Life Support, respectively. A catheter of suitable length may not be available immediately or the muscle structure of the chest wall may be modified in pneumothorax. Furthermore, alternative sites for needle thoracostomy and reference values of chest wall thickness (CWT) should be explored and warranted.

**Method:**

CT scan data and medical data of 650 eligible patients from October 2016 to December 2016 were reviewed. CWT values at four ICSs as well as four variables, namely, age, weight, height, and body mass index (BMI) for both men and women were compared using a nonparametric method, namely, the Wilcoxon signed-rank test. The associations between CWT and the four variables were assessed using the Pearson correlation coefficient. The overall performance of BMI, weight, and height in predicting CWT > 5 cm was evaluated using the receiver-operating characteristic (ROC) curve. Finally, the prediction models were built by using the bootstrap method.

**Results:**

Four variables, namely, age, height, weight, and BMI, were compared between the men and women groups. All four variables differed significantly between the two groups, and CWTs at all ICSs, except for the 3rd ICS, differed significantly between the two groups. Among the women, the area under the ROC curve (AUROC) of BMI for predicting CWT > 5 cm at 2nd ICS was larger than the AUROC of weight and height. Among the men, the AUROC of weight for predicting CWT > 5 cm at 2nd ICS was larger than that of BMI and height. The reference value tables were provided for five proposed models for women and men, respectively. Under emergencies, the variable, BMI, or even weight itself, could be used for predicting a failure performance of the needle decompression. For women, CWT at 5th ICS was predicted over 5 cm at BMI over 25.9 kg/m^2^ or weight over 103.1 kg. For men, CWT at 5th ICS was predicted over 5 cm at BMI over 25.5 kg/m^2^ or weight over 157.4 kg.

**Conclusion:**

Needle thoracostomy is the preferred first technique for many emergency providers for decompression. Therefore, a reference table for safe needle thoracostomy decompression at four usual sites, namely, 2nd ICS, 3rd CIS, 4th ICS, and 5th ICS, was recommended, which will enable paramedics and emergency specialists to rapidly determine CWT at the appropriate ICSs during emergencies.

## 1. Introduction

Tension pneumothorax, a life-threatening condition with a high mortality rate, frequently occurs in prehospital and emergency department settings [1, 2]. The incidence of tension pneumothorax is estimated to be 1%–5% in cases of major trauma [2–5]. Either needle thoracostomy or open finger thoracostomy is currently recommended interventions for decompression.

Needle thoracostomy, a lifesaving procedure, is easier to learn and faster to resuscitate patients than surgical decompression, and it converts a tension pneumothorax into a simple pneumothorax. Zengerink et al. found that the mean chest wall thickness (CWT) for either left- or right-sided measurement between men and women patients was significantly different [6]. Previous studies have shown that the average distance to be traversed during needle thoracostomy decompression performed at the 2nd intercostal space (ICS) differed between the patients from the United States (Ohio and California, 4.59 cm) and those from Japan (3.35 cm) [7–10]. This shows that patients from different ethnicities need different catheters to achieve satisfactory outcomes. In several studies, the use of a 5 cm catheter for decompression at the 2nd ICS resulted in failure rates in the range of 4%–50% [10–13]. Both Chang et al. and Aho et al. demonstrated an over 80% success rate for decompression with CWT measurements at the 2nd ICS in patients with an 8 cm catheter [14, 15]. The needle decompression failure rate with a routine 5 cm catheter at 5th ICS varied from 0% to 33% [10, 14, 16, 17].

Previously, a 5 cm long 14-gauge catheter was used, which was inserted into the 2nd ICS in the midclavicular line according to Advanced Trauma Life Support (ATLS) 9th Edition. In addition, the Tactical Combat Casualty Care Guidelines recommended that needle decompression would be performed either 5th ICS or 2nd ICS [18]. In early 2018, the American College of Surgeons published latest version of ATLS, i.e., the 10th edition; in this edition, the site for needle thoracostomy tension pneumothorax was changed from the 2nd ICS midclavicular line to the 5th ICS midaxillary line for adults [19].

Trauma has always been a very common and crucial cause of mortality and morbidity. An emergency specialist or critical care specialist who is under high stress needs to respond rapidly to select a proper site and needle size for needle thoracostomy decompression. The possible locations for the needle decompression are 2nd ICS, 3rd ICS, 4th ICS, and 5th ICS. In this study, we aimed to evaluate the required depth for successful decompression. CWT was determined not only for varying BMI, but also for weights in men and women, respectively. The corresponding risk-value tables for a routine 5 cm catheter based on five linear regression models were provided for immediate use during emergencies.

## 2. Materials and Methods

### 2.1. Participates

Consecutive patients who presented to Shuang-Ho Hospital from October 2016 to December 2016 for any reason and underwent chest computed tomography (CT) were reviewed after study approval by the Taipei Medical University-Joint Institutional Review Board. The patients were included if they were aged 20–80 years. The patients were excluded if they were aged <20 years or had a history of injury to the ribs. Patient demographic data, such as age, height, and weight, were collected from their medical charts. Two radiologists who were blinded to the patient demographics and injury diagnoses reviewed the patients' chest CT scans, retrospectively. CWT was measured as midclavicular line at four locations, namely, the 2nd ICS, 3rd ICS, 4th ICS, and 5th ICS.

### 2.2. Statistical Analysis

Four variables, namely, age, height, weight, and body mass index (BMI), were discussed in this study. BMI is calculated as weight in kilograms (kg) divided by the square of height in meters (m). The differences in the four variables between the women and men were assessed using a nonparametric method, namely, Wilcoxon signed-rank test, for nonnormally distributed data. The correlations between CWT and four variables were measured using the Pearson correlation coefficient.

The area under the receiver-operating characteristic (AUROC) curve was calculated for each variable to determine the discrimination performance of the four variables [20]. Five models evaluated in this study are as follows:

  Model 1: age + height + weight
  Model 2: age + BMI
  Model 3: age + weight
  Model 4: BMI
  Model 5: weight

Model 1 was a complete model consisting of all three related variables, namely, age, height, and weight. The first three models (Models 1–3) included the variable age, but only Model 1 included the variable height. Models 2 and 4 analyzed the variable BMI; however, Model 2 additionally considered the variable age. Models 3 and 5 assessed the variable weight; however, Model 3 additionally considered the variable age. Thousand random bootstrap resamples were used to construct prediction models for CWT [21]. The statistical significance level was set at *p* < 0.05, and all analyses were conducted in the R software (version 3.5.2).

## 3. Results

### 3.1. Characteristics

**Figure 1:**
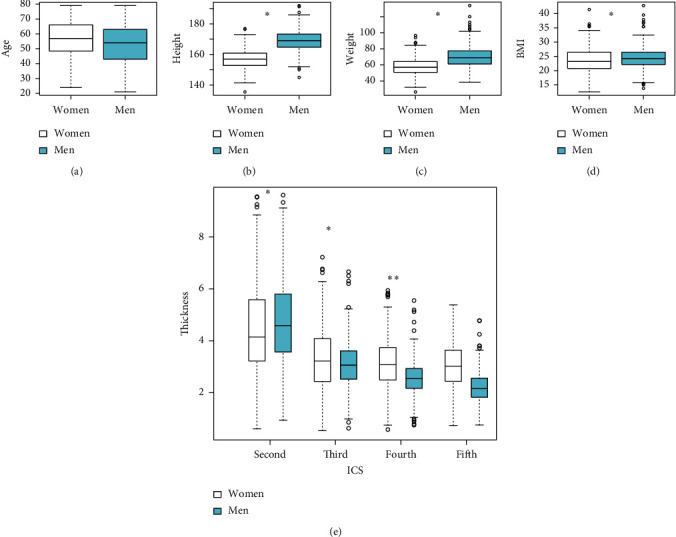
Participant characteristics (women (*N* = 268); men (*N* = 382)); ^*∗*^significant difference between women and men. (a) Age. (b) Height. (c) Weight. (d) BMI. (e) CWT.

In total, 650 chest CT scans (268 women and 382 men) were identified for review. The mean, minimum, median, and maximum of patient characteristics for both women and men are presented in Figure 1. In addition, the percentage of patients with CWT > 5 cm is shown in Table 1 for both women and men. The mean ages, 56.38 and 53.15 years, differed significantly between the women and men, respectively. The other three variables, namely, height, weight, and BMI, also differed significantly between the women and men. The mean of all four variables were smaller among the women than those among the men. CWT at all ICSs, except for the 3rd ICS, differed significantly between the men and women. The mean CWT at the 2nd ICSs among the women was smaller than that among the men (4.49 cm vs. 4.73 cm). However, the mean CWT at the other three ICSs were greater among the women than among the men. The maximum values of CWT at the 2nd ICS among the women and men exceeded 5 cm (women: 9.55 cm; men: 9.99 cm), but none of men had CWT > 5 cm at 5th ICS (maximum CWT of women: 5.38 cm; maximum CWT of men: 4.78 cm). The percentage of women participants with CWT larger than 5 cm at 2nd ICS, 3rd ICS, 4th ICS, and 5th ICS for women was 33.21%, 7.84%, 3.36%, and 0.37%, respectively. The percentage of men participants with CWT larger than 5 cm at 2nd ICS, 3rd ICS, 4th ICS, and 5th ICS was 40.58%, 2.62%, 0.79%, and 0%, respectively. Between women and men, there were more male participates with larger than 5 cm CWT at 2nd ICS, but none of men with larger than 5 cm CWT at 5th ICS.

### 3.2. Correlations

Correlation analysis assessing the relationships between the four ICSs and the four predictors revealed moderate significant correlations for all, except for height, which was not significantly correlated with any of the four ICSs among the women (*p* > 0.05). The results of correlation coefficients are shown in Table 2. The predictor age was significantly negatively correlated with all the four ICSs, and the correlation coefficient was smaller for the 5th ICS than for the other three ICSs among the women and men. Comparing the BMI-CWT relationship with the weight-CWT relationship, BMI was more strongly related with CWT than was weight at the four ICSs among the women; however, weight was more strongly correlated with CWT than was BMI at the 2nd ICS among the men.

### 3.3. The ROC Curve of the Variables

**Figure 2:**
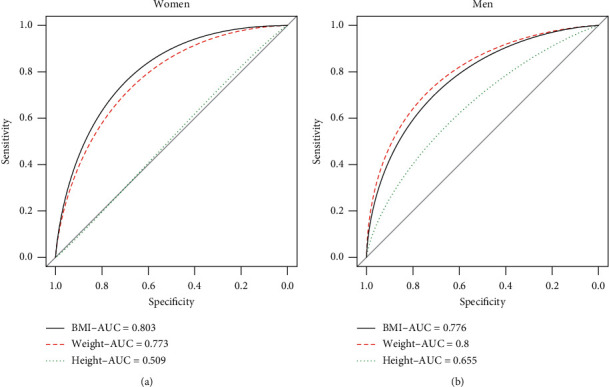
ROC curve of BMI (solid line), weight (dashed line), and height (dotted line). Left panel: female; right panel: male. AUC: area under the curve.

A CWT value of >5 cm was considered as a risk value for needle decompression at the any ICS. The ROC curves of three variables, namely, weight, height, and BMI, were compared for predicting the failure (CWT > 5 cm) at the 2nd ICS (Figure 2). The left and right panels show the ROC curves in the women and men, respectively. According to the area under the ROC curve (AUROC), the AUROC of BMI (0.8) was the largest among the three variables among the women. However, the AUROC of weight (0.8) was the largest among the men. Thus, BMI was needed for determining CWT at the 2nd ICS among the women, whereas the weight was sufficient for determining CWT among the men. Demographic differences were observed between the variables and ICSs; therefore, the predictor models were evaluated for the men and women separately in the following sections. Height did not perform satisfactorily as a predictor among either the women or men; therefore, it was not considered as a strong predictor for CWT.

### 3.4. Comparison of Proposed Models

Five proposed models were evaluated for men and women separately. *R*^2^ represents model performance, and the estimates of the each variable are shown in Table 3 and 4. Overall, the performance of the five models in predicting CWT was more satisfactory among the men than among the women. A comparison of the four ICSs revealed that the performance of the five models in predicting CWT was the highest at the third ICS. Clearly, among the five models, the complete model, namely, Model 1, consisting of age, weight, and height, exhibited the highest performance in predicting CWT. The suggestion risk values for both women and men at 5th ICS are as follows.

#### 3.4.1. Weight Suggestion for Model 1

The estimate of CWT in Model 1 for 5th ICS was calculated as follows (from Table 3):

 
7.182 − 0.014*∗*age+0.052*∗*weight − 0.041*∗*height, for women
 
4.029 − 0.002*∗*age+0.039*∗*weight − 0.026*∗*height, for men

**Figure 3:**
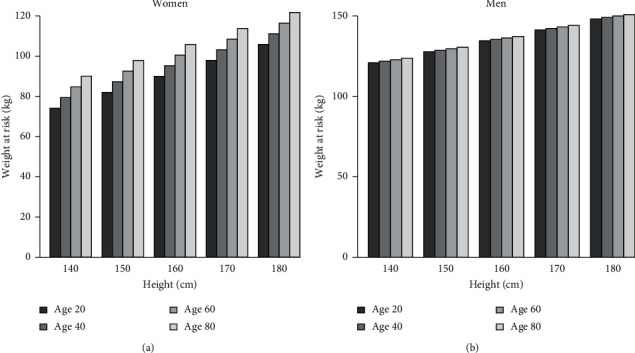
Risk weights for women (left panel) and for men (right panel) at 5th ICS from Model 1.

The table of the risk weight suggestion for Model 1 (Figure 3 and Table 5) is provided for quick reference. The values in Figure 3 are risk value of weights for different ages and heights when performing needle decompression. For example, a woman aged 60 years with height of 170 cm may have a failure of needle decompression with the weight exceeds 108.50 kg. For a man aged 20 years with a height of 160 cm may have a failure performance of needle decompression at 5th ICSs if his weight exceeds 134.53 kg, respectively. The reference table for the other sites of needle decompression is shown in Table 5.

#### 3.4.2. BMI Suggestion for Model 2 and Weight Suggestion for Model 3

The estimate of CWT for Model 2 at 5th ICS was calculated as follows:

 
0.702 − 0.013*∗*age+0.127*∗*BMI, for women
 
−0.419 − 0.003*∗*age+0.113*∗*BMI, for men

For the same aged women, as BMI increases one unit and the CWT increases 0.127 cm. For the same aged men, as BMI increases one unit and the CWT increases 0.113 cm. The estimate of CWT in Model 3 for 5th ICS was calculated as follows:

 
0.744 − 0.006*∗*age+0.044*∗*weight, for women
 
−0.247 − 0.003*∗*age+0.033*∗*weight, for men

As weight increases 1 kg, the CWT increases 0.044 cm and 0.033 cm for the same aged women and men, respectively. Model 2 consisted of age and BMI, whereas Model 3 consisted of age and weight. The BMI suggestion at 5th ICS is shown in the upper panel of Table 6 for Model 2, and weight suggestion at 5th ICS is shown in the bottom panel of Table 6 for Model 3. For a woman aged 50 years, CWT at the 5th ICS might be larger than 5 cm if her BMI exceeds 38.84 kg/m^2^. A man aged 70 years will be at risk of CWT > 5 cm at the 5th ICSs if his BMI exceeds 49.60 kg/m^2^. Form the results of Model 3, a woman age 30 years might have a failure needle decompression if her weight over 100.2 kg. The corresponding BMI chart and weight chart for the other site of needle decompression are shown in Table 7.

#### 3.4.3. BMI Suggestion for Model 4 and Weight Suggestion for Model 5

The estimate of CWT in Model 4 for 5th ICS was calculated as follows:

 
0.057+0.124*∗*BMI, for women
 
−0.615+0.115*∗*BMI, for men

As BMI increases one unit, the CWT increases 0.124 cm and 0.115 cm for women and men, respectively. The estimate of CWT in Model 5 for 5th ICS was calculated as follows:

 
0.387+0.045*∗*weight, for women
 
−0.049+0.032*∗*weight, for men

**Figure 4:**
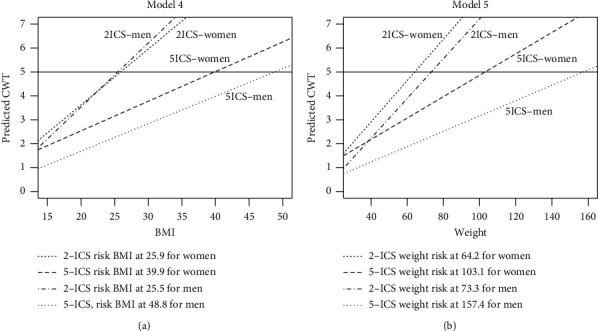
The estimation of CWT via Model 4 (left panel) and Model 5 (right panel). Women at 2nd ICS: dashed line; women at 5th ICS: long dash line; men at 2nd ICS: dotted line; men at 5th ICS: dotted line.

As weight increases 1 kg, the CWT increases 0.045 cm and 0.032 cm for women and men, respectively. The BMI suggestion in Model 4 and the weight suggestion for Model 5 are shown in the left panel and right panel of Figure 4, respectively. CWT at the 5th ICS will exceed 5 cm if BMI exceeds 39.9 kg/m^2^ and 48.8 kg/m^2^ among the women and men, respectively. From the result of Model 5, CWT > 5 cm will be observed at the 5th ICSs if the weight exceeds 103.1 kg and 157.4 kg among the women and men, respectively.

## 4. Discussion

By using a bootstrapping method, we showed that the predicted length of the catheter for decompression at four possible sites, namely, 2nd ICS, 3rd ICS, 4th ICS, and 5th ICS, among women and men, respectively. During emergencies, sometimes, personal information may not be sufficient for accurate estimation of CWT. In this study, we proposed five predicting models, ranging from simple to complex and from approximate to accurate estimate CWT in men and women, respectively.

Selecting the safest ICS is a major challenge for emergency specialists. As described by ATLS, needle placement is suggested at the 5th ICS instead of the 2nd ICS for adults in early 2018. Inadequate length of catheters may result in risk of pleural bleeding, pulmonary artery injury, and cardiac tamponade [22, 23]. Previous studies have confirmed that the failure rate of needle decompression was lower at the 5th ICS than at the 2nd ICS [9–13, 24]. The 5th ICS is the primary site to attempt needle decompression according to the recent guideline, but this site sometime is not available for trauma patients. Therefore, the estimations of CWT at alternative sites, such as 3rd ICS or 4th ICS, are needed. In this study, the four prediction models and its estimators were provided for women and men, respectively. In addition, the risk values for the 5th ICS were provided and the risk values for the other sites were provided as a supplement.

In this study, we evaluated the association between mean CWT and three variables via the ROC curve. We found that BMI was the best predictor among the three variables in the women to determine CWT with an AUROC of 0.803 and weight was the second strong variable to predict CWT over 5 cm with an AUROC of 0.77. On the other hand, weight was the best predictor among the three variables for CWT for the men with an AUROC of 0.8. According to the result of bootstrapping estimators for Model 4, the risk values of BMI are 39.9 kg/m^2^ and 48.8 kg/m^2^ for women and men, respectively. Moreover, the risk values of weight from the result of Model 5 were 103.1 kg and 157.4 kg, respectively.

CT is the most common and accurate used tool for measuring CWT, but our study was limited by its retrospective nature and a possible bias. The measurement bias was minimized by blinding the radiologists who independently reviewed the patients' CT data. Consecutive patients were sampled to avoid a sampling bias. However, some unmeasurable images (owing to organ injury or bone injury) and exclusion of images may have introduced a bias in the results.

## 5. Conclusion

Needle thoracostomy is a lifesaving procedure performed to change a tension pneumothorax to a normal pneumothorax, but it is related with potentially serious complications. The major problem regarding the use of NT is the difficulty in determining an adequate catheter length which is related to the patient's chest wall thickness. In this study, CWT was estimated using CT-based analysis along with the bootstrapping method. BMI and weight were found as the best predictors of CWT among the women and men, respectively. For women, the performance of needle decompression would be safe at the 5th ICS (CWT < 5 cm) if her BMI is less than 39.9 kg/m^2^ or her weight is less than 103.1 kg. For men, the performance of needle decompression would be safe at the 5th ICS (CWT < 5 cm) if his BMI is less than 48.8 kg/m^2^ or his weight is less than 157.4 kg.

## Figures and Tables

**Table 1:** Participant characteristics.

	Women (*N* = 268)					Men (*N* = 382)					*p* value
Variable	Min	Med	Mean	Max		Min	Med	Mean	Max		
Age	24.00	57.00	56.38	79.00		21.00	54.00	53.15	79.00	—	<0.01
Height	135.50	157.00	159.74	176.90		145.00	169.00	168.93	191.90		<0.01
Weight	26.40	57.15	58.25	96.60		38.50	69.00	70.09	133.80	—	<0.01
BMI	12.56	23.17	23.70	41.43		13.81	24.24	24.48	42.81		<0.01
ICS	Min	Med	Mean	Max	>5 cm (%)	Min	Med	Mean	Max	>5 cm (%)	*p* value
2nd ICS	0.61	4.15	4.49	9.55	33.21%	0.94	4.59	4.73	9.99	40.58%	0.03
3rd ICS	0.54	3.22	3.29	7.22	7.84%	0.63	3.06	3.09	6.66	2.62%	0.06^+^
4th ICS	0.58	3.08	3.10	5.94	3.36%	0.75	2.54	2.55	5.55	0.79%	<0.01
5th ICS	0.74	3.02	2.99	5.38	0.37%	0.76	2.16	2.20	4.78	0%	<0.01

*N*: sample size; Min: minimum; Med: median; Max: maximum; BMI: body weight index; *p* value: difference between women and men; ^+^nonsignificant.

**Table 2:** Correlation coefficients between variables and ICSs.

Variable	Women				Men			
	2nd ICS	3rd ICS	4th ICS	5th ICS	2nd ICS	3rd ICS	4th ICS	5th ICS
Age	−0.195	−0.190	−0.222	−0.129	−0.422	−0.392	−0.288	−0.181
Height	0.023^+^	0.013^+^	0.038^+^	0.006^+^	0.318	0.304	0.236	0.188
Weight	0.566	0.624	0.603	0.569	0.663	0.769	0.749	0.731
BMI	0.591	0.660	0.629	0.608	0.622	0.755	0.764	0.764

*N*: sample size; sd: standard deviation; BMI: body weight index; *p* value: difference between two means; ^+^nonsignificant.

**Table 3:** *R* ^2^ of proposed models.

	Women				Men			
	2nd ICS	3rd ICS	4th ICS	5th ICS	2nd ICS	3rd ICS	4th ICS	5th ICS
Model 1	0.408	0.492	0.463	0.391	0.504	0.654	0.617	0.593
Model 2	0.405	0.492	0.468	0.399	0.494	0.647	0.613	0.587
Model 3	0.338	0.404	0.389	0.325	0.486	0.614	0.562	0.535
Model 4	0.346	0.433	0.393	0.368	0.385	0.569	0.583	0.583
Model 5	0.318	0.387	0.361	0.321	0.438	0.591	0.560	0.533

Model 1 consisted of three variables, namely, age, height, and weight. Model 2 consisted of two variables, namely, age and BMI. Model 3 consisted of two variables, namely, age and weight. Model 4 consisted of the variable BMI. Model 5 consisted of the variable weight.

**Table 4:** Bootstrapping coefficients of five proposed models.

		Women					Men		
		2nd ICS	3rd ICS	4th ICS	5th ICS	2nd ICS	3rd ICS	4th ICS	5th ICS
Model 1	*R* ^2^	0.408	0.492	0.463	0.391	0.504	0.654	0.617	0.593
	Intercept	13.581	10.447	8.687	7.182	8.101	5.837	4.921	4.029
	Age	−0.036	−0.026	−0.024	−0.014	−0.037	−0.017	−0.008	−0.002
	Weight	0.100	0.079	0.063	0.052	0.083	0.057	0.044	0.039
	Height	−0.082	−0.066	−0.050	−0.041	−0.043	−0.035	−0.030	−0.026
Model 2	*R* ^2^	0.405	0.492	0.468	0.399	0.494	0.647	0.613	0.587
	Intercept	0.648	0.089	0.729	0.702	0.992	0.033	−0.116	−0.419
	Age	−0.034	−0.024	−0.022	−0.013	−0.043	−0.020	−0.009	−0.003
	BMI	0.242	0.193	0.154	0.127	0.245	0.168	0.129	0.113
Model 3	*R* ^2^	0.338	0.404	0.389	0.325	0.486	0.614	0.562	0.535
	Intercept	0.698	0.155	0.775	0.744	1.143	0.229	0.085	−0.247
	Age	−0.020	−0.013	−0.014	−0.006	−0.030	−0.011	−0.003	0.003
	Weight	0.085	0.067	0.054	0.044	0.074	0.049	0.037	0.033
Model 4	*R* ^2^	0.346	0.433	0.393	0.368	0.385	0.569	0.583	0.583
	Intercept	−0.049	−1.125	−0.405	0.057	−1.806	−1.269	−0.715	−0.615
	BMI	0.234	0.186	0.148	0.124	0.267	0.178	0.133	0.115
Model 5	*R* ^2^	0.318	0.387	0.361	0.321	0.438	0.591	0.560	0.533
	Intercept	−0.553	−0.676	−0.090	0.387	−1.092	−0.624	−0.125	−0.049
	Weight	0.087	0.068	0.055	0.045	0.083	0.053	0.038	0.032

Model 1 consisted of three variables, namely, age, height, and weight. Model 2 consisted of two variables, namely, age and BMI. Model 3 consisted of two variables, namely, age and weight. Model 4 consisted of the variable BMI. Model 5 consisted of the variable weight.

**Table 5:** Weight chart for Model 1; women: left panel; men: right panel.

Age	ICS	Women height					Men height				
		140	150	160	170	180	140	150	160	170	180
20	2nd	36.44	44.68	52.91	61.15	69.38	43.76	48.91	54.06	59.21	64.36
	3rd	53.93	62.24	70.54	78.85	87.16	76.11	82.17	88.22	94.28	100.33
	4th	61.25	69.28	77.31	85.34	93.38	100.22	106.99	113.77	120.56	127.33
	5th	74.13	82.05	89.98	97.91	105.84	120.95	127.74	134.53	141.32	148.11
40	2nd	43.69	51.92	60.16	68.39	76.62	52.59	57.75	62.90	68.05	73.20
	3rd	60.54	68.85	77.16	85.47	93.78	82.10	88.16	94.21	100.26	106.32
	4th	68.81	76.84	84.87	92.90	100.93	103.75	110.53	117.31	124.09	130.86
	5th	79.42	87.35	95.28	10321	111.14	121.87	128.66	135.45	142.24	149.03
60	2nd	50.93	59.17	67.41	75.64	83.88	61.43	66.58	71.73	76.88	82.03
	3rd	67.16	75.47	83.78	92.09	100.40	88.08	94.14	100.19	106.25	112.30
	4th	76.38	84.41	92.44	100.47	108.50	107.28	114.06	120.84	127.62	134.39
	5th	84.72	92.65	100.58	108.50	116.43	122.79	129.58	136.37	143.16	149.95
80	2nd	58.18	66.42	74.65	82.89	91.12	70.27	75.42	80.57	85.72	90.87
	3rd	73.78	82.09	90.40	98.71	107.02	94.07	100.12	106.18	112.23	118.29
	4th	83.94	91.97	99.99	108.03	116.06	110.81	117.59	124.37	131.15	137.92
	5th	90.01	97.94	105.87	113.80	121.73	123.71	130.50	137.29	144.08	150.87

Model 1 consisted of three variables, namely, age, height, and weight. H: height (cm); W: weight (kg).

**Table 6:** BMI chart for women and men at 5th ICS with a failure performance for Model 2 (upper panel); weight chart for women and men at 5th ICS with a failure performance for Model 3 (lower panel).

Model 2: age + BMI							
BMI	Age (year)						
	20	30	40	50	60	70	80
Women	35.82	36.82	37.83	38.84	39.84	40.85	41.86
Men	48.28	48.55	48.81	49.07	49.34	49.60	49.86

Model 3: age + weight							
Weight	Age (year)						
	20	30	40	50	60	70	80
Women	98.89	100.2	101.5	102.8	104.1	105.4	106.7
Men	157.8	157.0	156.2	155.4	154.6	153.8	153.0

Model 2 consisted of two variables, namely, age and BMI. Model 3 consisted of two variables, namely, age and weight.

**Table 7:** BMI chart for Model 2 and weight chart for Model 3.

*Model 2: age + BMI*														
	Women age (years)							Men age (years)						
ICS	20	30	40	50	60	70	80	20	30	40	50	60	70	80
2nd	20.76	22.15	23.55	24.94	26.33	27.72	29.11	19.81	21.55	23.29	25.02	26.76	28.50	30.23
3rd	28.01	29.26	30.51	31.77	33.02	34.27	35.52	31.96	33.14	34.32	35.51	36.69	37.87	39.05
4th	30.71	32.17	33.63	35.10	36.56	38.02	39.49	41.14	41.85	42.56	43.27	43.97	44.68	45.39
5th	35.82	36.82	37.83	38.84	39.84	40.85	41.86	48.28	48.55	48.81	49.07	49.34	49.60	49.86

*Model 3: age + weight*														
ICS	Women age (years)							Men age (years)						
	20	30	40	50	60	70	80	20	30	40	50	60	70	80

2nd	55.63	58.02	60.41	62.81	65.20	67.59	69.98	60.38	64.41	68.43	72.46	76.49	80.51	84.54
3rd	76.46	78.47	80.48	82.49	84.50	86.51	88.52	101.2	103.5	105.8	108.1	110.4	112.7	115.0
4th	84.16	86.78	89.39	92.00	94.62	97.23	99.84	133.3	134.0	134.7	135.5	136.2	137.0	137.7
5th	98.89	100.2	101.5	102.8	104.1	105.4	106.7	157.8	157.0	156.2	155.4	154.6	153.8	153.0

Model 2 consisted of two variables, namely, age and BMI. Model 3 consisted of two variables, namely, age and weight.

## Data Availability

The data used to support the findings of this study are available from the corresponding author upon request.
